# Anabolic–Androgenic Steroids Revisited: Structural Biology, Receptor Signaling, and Mechanisms of Anabolic–Androgenic Dissociation

**DOI:** 10.3390/ijms27062581

**Published:** 2026-03-11

**Authors:** Magdalena Wiacek, Igor Z. Zubrzycki

**Affiliations:** Department of Medical and Health Sciences, Casimir Pulaski Radom University, Chrobrego 27, 26-600 Radom, Poland

**Keywords:** anabolic–androgenic steroids, androgen receptor, structure–activity relationships, steroid hormone signaling, non-genomic signaling, C17 substitution

## Abstract

Steroid hormones exert diverse and tissue-specific biological effects despite sharing a conserved tetracyclic scaffold. Among these, anabolic–androgenic steroids (AAS) present a longstanding paradox: structurally related compounds can elicit markedly different anabolic, androgenic, and cardiovascular outcomes. This narrative review integrates advances in steroid structural chemistry, androgen receptor (AR) biology, and intracellular signaling to elucidate the molecular mechanisms underlying anabolic–androgenic dissociation. We summarize classical genomic and emerging non-genomic modes of steroid action, emphasizing how receptor conformation, ligand-binding domain architecture, co-regulator recruitment, and signaling bias shape downstream biological responses. Particular focus is placed on the structure–activity relationships of endogenous and synthetic androgens, with C17-substitution chemistry highlighted as a central determinant of receptor affinity, metabolic stability, pharmacokinetics, and tissue selectivity. By linking molecular structure to receptor-level mechanisms, we contextualize the physiological and pathophysiological effects of major AAS classes used clinically and non-medically, including testosterone esters, 19-nor derivatives, 17α-alkylated steroids, heterocyclic compounds, and halogenated compounds. While much of the mechanistic evidence derives from preclinical models, the integrated framework presented here provides a coherent basis for interpreting divergent anabolic, androgenic, and cardiovascular effects observed in humans. Collectively, this review bridges fundamental steroid biology with applied physiology and sports medicine, offering mechanistic insight relevant to therapeutic development, anti-doping science, and risk assessment of supraphysiological androgen exposure.

## 1. Introduction

### 1.1. Steroid Hormone Biology

Steroid hormones represent a fundamental class of signaling molecules that orchestrate complex physiological processes across virtually all organ systems. Derived from cholesterol, these lipophilic hormones include five major classes: glucocorticoids, mineralocorticoids, estrogens, androgens, and progestins [1,2]. Each class exerts powerful effects on target tissues through interactions with specific receptor proteins, influencing processes ranging from reproductive function and metabolic homeostasis to immune regulation and neural development [2]. Importantly, the biological effects of steroid hormones are not uniform across tissues but depend on cell type, developmental stage, and the prevailing physiological and metabolic environment, resulting in highly context-dependent signaling outcomes.

The mechanisms by which steroid hormones exert their diverse effects have been the subject of intensive investigation for over four decades. Initially, steroid hormone action was understood primarily through the genomic signaling paradigm, in which hormones diffuse across the plasma membrane and bind to intracellular nuclear receptors that function as ligand-dependent transcription factors [3]. Upon ligand binding, these receptors undergo conformational changes, translocate to the nucleus, and regulate gene expression by interacting with specific hormone response elements in target genes. This classical genomic mechanism accounts for many of the long-term and tissue-specific effects of steroid hormones, including differentiation, growth, and metabolic regulation.

Over the past two decades, however, accumulating evidence has revealed a more complex and integrative model of steroid hormone signaling. In addition to their well-characterized genomic actions, steroid hormones are now known to elicit rapid, non-genomic effects mediated by membrane-associated receptors and extranuclear signaling pathways [4,5,6]. These non-genomic actions occur on timescales of seconds to minutes and involve the activation of intracellular signaling cascades such as kinase pathways, ion fluxes, and second messenger systems. Such mechanisms allow steroid hormones to modulate cellular function independently of direct transcriptional regulation and to respond dynamically to acute physiological demands.

Crucially, genomic and non-genomic steroid signaling should not be viewed as separate or competing mechanisms, but rather as interconnected components of a unified signaling network. Crosstalk between extranuclear signaling pathways and nuclear receptor activity enables fine-tuning of transcriptional responses, modulation of receptor sensitivity, and integration of hormonal signals with other regulatory inputs, including mechanical, metabolic, and inflammatory cues. As a result, the ultimate biological outcome of steroid hormone action reflects not only ligand structure and receptor affinity but also the cellular activation state, availability of coregulatory proteins, chromatin accessibility, and the broader physiological context in which signaling occurs.

This multilayered signaling architecture provides the mechanistic foundation for the diverse, tissue-specific, and sometimes divergent effects of steroid hormones observed across different biological systems. Understanding these integrated signaling principles is essential for interpreting how structurally related steroid compounds can produce markedly distinct biological responses. It also sets the stage for subsequent discussion of receptor selectivity, structure–activity relationships, and context-dependent steroid action in applied physiological settings.

### 1.2. Genomic vs. Non-Genomic Signaling

Genomic actions primarily involve the binding of steroids to their respective nuclear receptors. This interaction typically alters gene transcription, thereby affecting protein synthesis and cellular function. For instance, steroid hormone receptors, such as the estrogen receptor (ER), translocate to the nucleus upon ligand binding, where they initiate transcription of genes involved in growth and differentiation [7]. The classical model of steroid receptor action emphasizes that steroid-receptor complexes function as transcription factors. They typically bind to hormone response elements (HREs) in the promoters of target genes [8]. For instance, the estrogen receptor (ER) mediates the effects of estrogens by promoting the transcription of genes involved in reproductive function and cellular proliferation [9]. Studies have shown that the glucocorticoid receptor (GR) similarly regulates a broad range of genes involved in stress responses and inflammation through its binding to glucocorticoid response elements in target genes [10,11]. This model highlights the significance of chromatin accessibility, which dictates the binding patterns of these receptors to DNA and ultimately determines the target genes for transcriptional regulation [10].

Interestingly, recent research has uncovered a more intricate picture of steroid signaling, emphasizing that its effects extend beyond established genomic pathways. While the direct effects of steroid hormones on gene expression have been well characterized, evidence increasingly suggests complex interactions and integrative mechanisms for regulating transcription and chromatin structure. Specifically, transcription factor cooperation, chromatin remodeling, and the roles of pioneer factors are pivotal in determining how specific genes are expressed in response to steroid hormones [11,12].

Nongenomic actions of steroids involve rapid effects that occur independently of changes in gene expression. These actions typically manifest within seconds to minutes and hinge on the interaction of steroids with membrane-bound receptors or signaling pathways. A noteworthy finding is the existence of rapid actions mediated by steroid hormone receptors localized at the plasma membrane. For instance, studies have demonstrated that both estrogen and aldosterone can activate endothelial nitric oxide synthase (eNOS) via membrane-associated receptors, leading to immediate vascular responses [13,14]. This illustrates how steroids can acutely influence cardiovascular function without entering the nucleus.

Steroids engage in nongenomic signaling via several pathways that often involve G-protein-coupled receptors (GPCRs) and downstream signaling cascades such as the phosphatidylinositol 3-kinase (PI3K) pathway and mitogen-activated protein kinases (MAPKs) [15,16]. For example, the identification of GPR30 as a receptor that mediates some of estrogen’s rapid effects has highlighted the role of GPCRs in steroid hormone signaling [17,18]. These effects are crucial for various physiological processes, including metabolism, cardiovascular function, and cellular proliferation.

Moreover, the actions of sex steroid hormones, including estrogens and androgens, have been shown to initiate signaling cascades that modulate cellular responses, such as calcium influx and kinase activation, independent of traditional transcriptional mechanisms [18,19]. Such pathways have been observed in diverse cell types, demonstrating the versatility of steroid action across different tissues.

Importantly, many synthetic anabolic–androgenic steroids display differential activation of genomic versus non-genomic androgen receptor pathways. These biases may contribute to rapid hypertrophic signaling, altered vascular responses, and cardiovascular risk profiles observed with supraphysiological AAS use.

At the molecular level, intracellular pathways drive androgen receptor function and, as such, steroid androgenic properties. Specifically, exosomal miR-222-3p has been shown to enhance mTOR signaling, promoting CRPC progression and underscoring the regulatory complexity that influences both anabolic and androgenic state transitions [20]. The roles of different integrins and the transition from androgen-dependent to androgen-independent phenotypes significantly contribute to cellular morphology [21,22].

### 1.3. Scope and Aims

Despite decades of clinical, experimental, and forensic investigation, the molecular basis underlying the dissociation between anabolic and androgenic effects of steroid hormones remains incompletely resolved. In particular, it is still unclear how anabolic–androgenic steroids (AAS) that share a conserved steroid scaffold can produce markedly divergent, tissue-specific biological outcomes. This unresolved issue remains a central challenge in endocrinology, pharmacology, and sports medicine, with important implications for therapeutic development, cardiovascular risk assessment, and anti-doping science.

The primary aim of this narrative review is to integrate principles of steroid structural chemistry with contemporary insights into androgen receptor (AR) biology and intracellular signaling to elucidate how specific molecular modifications influence receptor engagement, signaling bias, tissue selectivity, and pharmacokinetic behavior. Rather than cataloguing individual compounds exhaustively, the review focuses on mechanistic relationships that link chemical structure to biological function.

Accordingly, the scope of this review is centered on anabolic–androgenic steroids, examined through four complementary dimensions: (1) structural features of the steroid nucleus and key sites of chemical modification; (2) androgen receptor structure, ligand recognition, and genomic versus non-genomic signaling mechanisms; (3) structure–activity relationships governing anabolic–androgenic dissociation; and (4) physiological and pathophysiological consequences of AAS exposure in clinical and athletic contexts.

Special emphasis is placed on AAS action within the physiological milieu of athletic training, where mechanical loading, metabolic stress, and neuroendocrine adaptation may substantially modify steroid signaling outcomes. While genomic and non-genomic mechanisms of steroid hormone action are introduced to provide essential conceptual grounding, the overarching objective is to connect molecular structure to receptor-level mechanisms and performance-relevant biological effects. By doing so, this review aims to provide an integrated framework that bridges fundamental steroid biology with applied sports medicine and anti-doping perspectives.

### 1.4. Literature Search Strategy

This narrative review was informed by a structured literature search conducted in PubMed, Scopus, and Web of Science using combinations of the following keywords: steroid hormones, androgen receptor, ligand-binding domain, anabolic–androgenic steroids, structure–activity relationship, C17 substitution, genomic and non-genomic signaling, and sports doping. Priority was given to peer-reviewed articles published in English, including structural biology studies, mechanistic in vitro and in vivo investigations, clinical trials, and authoritative reviews. Where appropriate, historical sources were included to contextualize the evolution of anabolic steroid development. Case reports and non-peer-reviewed sources were excluded unless directly relevant to anti-doping detection or forensic identification. The objective was not exhaustive coverage of all compounds, but a mechanistic representation of key structural classes.

## 2. Structural Biology of Steroid Hormones

Understanding receptor-level determinants is essential for interpreting how structural steroid modifications translate into divergent anabolic, androgenic, and cardiovascular outcomes, particularly under conditions of athletic training and misuse. Moreover, by integrating structural chemistry with receptor biology and signaling mechanisms, this review provides a unifying framework for understanding how anabolic–androgenic steroids with closely related scaffolds can elicit profoundly different biological and performance-related effects.

### 2.1. Steroid Nucleus Overview

**Figure 1:**
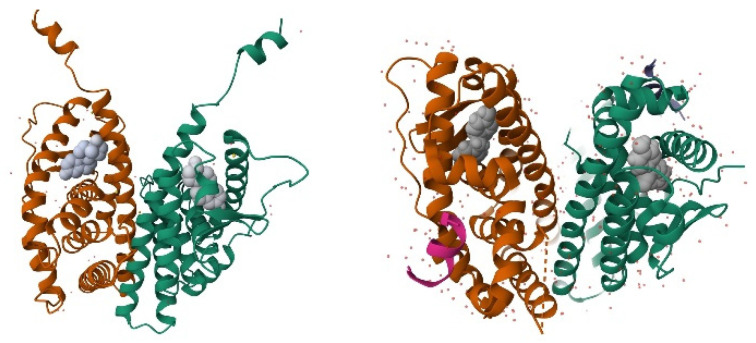
Three-dimensional structures of estrogen receptor α (ERα) and estrogen receptor β (ERβ) ligand-binding domains in complex with an estrogenic ligand. The receptors are shown in ribbon representation, highlighting the conserved α-helical fold characteristic of nuclear hormone receptors, with the bound ligand depicted in space-filling representation. Structural differences between ERα and ERβ contribute to subtype-specific ligand recognition and signaling.

Steroid hormones comprise five major classes, each with distinct physiological roles and specific receptor proteins. Class 1: Estrogens, mediated primarily by estrogen receptors alpha and beta (ERα and ERβ), regulate reproductive function, bone metabolism, and cardiovascular health [2,23] (Figure 1).

**Figure 2:**
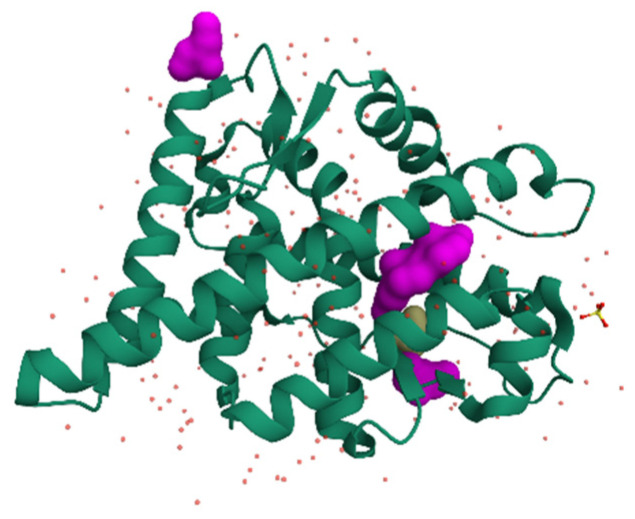
Crystal structure of the human androgen receptor ligand-binding domain (AR-LBD) in complex with testosterone. The receptor is shown in ribbon representation, highlighting the canonical α-helical fold of nuclear hormone receptors, while testosterone is depicted in surface representation within the ligand-binding pocket. Key hydrophobic and polar interactions stabilize ligand binding and promote the active receptor conformation.

Class 2: Androgens, acting through the androgen receptor (AR), control male sexual development and function, as well as muscle and bone mass in both sexes [2,24]. The structure of the androgen receptor (AR) is shown in Figure 2.

Class 3: Progestins, which signal through progesterone receptors (PR-A and PR-B), are essential for female reproductive physiology, particularly for pregnancy maintenance [2,25].

Class 4: Glucocorticoids bind to the glucocorticoid receptor (GR) and regulate metabolism, immune function, and stress responses [2,26]. Class 5: Mineralocorticoids, acting through the mineralocorticoid receptor (MR), control electrolyte homeostasis and blood pressure [2,27].

### 2.2. Estrogens vs. Androgens

**Figure 3:**
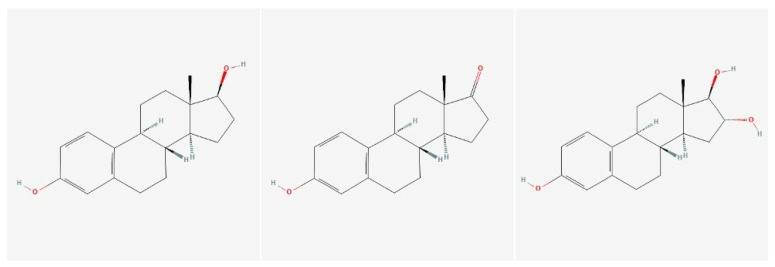
Structural comparison of endogenous estrogens highlighting the conserved steroid framework and aromatic A-ring. Variations in functional groups at C16 and C17 account for the distinct biological activities of estradiol, estrone, and estriol.

Core structural features of estrogens comprise four fused rings—A, B, C (six-membered) and D (five-membered)—lack of a C-19 methyl group, an aromatic A-ring, a -OH group at C3 of the A-ring (critical for binding to estrogen receptors) and a hydroxyl or keto group at C17; Estradion ⟶ -OH, Estrone ⟶ =O, and Estriol ⟶ -OH at C16 and C17 (Figure 3).

Core structural features of androgens comprise four fused rings—A, B, C (six-membered), and D (five-membered)—the presence of an angular C-19 methyl group, a non-aromatic A-ring typically containing a $\Delta^{4}$ double bond, a keto group at C3 of the A-ring (critical for binding to androgen receptors) and substitutions at C17, including hydroxyl, keto, alkyl, esterified, ether, or bulky synthetic groups, which critically modulate androgen receptor affinity, metabolic stability, and pharmacokinetics. The concise description of androgenic steroids resulting from C17 substitution is presented in Table 1.

These classical nuclear receptors belong to the nuclear receptor superfamily of ligand-dependent transcription factors, sharing a common structural organization with distinct functional domains [2,28]. The N-terminal domain contains activation functions, the central DNA-binding domain recognizes specific hormone response elements, and the C-terminal ligand-binding domain confers hormone specificity and recruits coregulatory proteins [5,27]. Beyond these well-characterized nuclear receptors, recent research has identified membrane-associated receptors for each steroid class, including G protein-coupled estrogen receptor (GPER), membrane progesterone receptors (mPRs), and putative membrane androgen receptors [5,25,29].

### 2.3. Receptor Structure and Ligand Recognition

The nuclear receptor superfamily is a diverse group of ligand-activated transcription factors characterized by modular structures that support diverse functions in gene regulation. These receptors share a characteristic modular structure comprising several domains that contribute to their functional diversity. The key domains include the N-Terminal Domain (NTD), DNA-Binding Domain (DBD), hinge region, Ligand-Binding Domain (LBD), and C-Terminal F Domain (F domain) [30,31]. The features of domain organization are shown in Table 1.

**Table 1:** Structural and functional domains of nuclear steroid hormone receptors. This table summarizes the canonical modular organization of nuclear steroid hormone receptors, including the N-terminal domain (NTD), DNA-binding domain (DBD), hinge region, ligand-binding domain (LBD), and the variable C-terminal F domain. For each domain, key structural characteristics and principal functional roles are outlined, highlighting mechanisms of ligand-independent and ligand-dependent transcriptional activation, DNA recognition via hormone response elements, nuclear localization, and co-regulator recruitment. Together, these domains provide the structural framework underlying receptor specificity, signaling versatility, and tissue-dependent steroid hormone action.

Receptor Domain	Key Structural Features	Primary Functional Role	References
N-Terminal Domain (NTD)	Contains activation function 1 (AF-1); intrinsically disordered region	Mediates ligand-independent transactivation and interacts with transcriptional co-regulators	[31,32,33]
DNA-Binding Domain (DBD)	Highly conserved; contains two zinc finger motifs	Recognizes and binds specific hormone response elements (HREs) in target gene promoters	[33,34]
Hinge Region	Flexible linker region; contains nuclear localization signals (NLS)	Facilitates nuclear translocation and provides conformational flexibility between DBD and LBD	[35]
Ligand-Binding Domain (LBD)	Contains activation function 2 (AF-2); hydrophobic ligand-binding pocket	Binds steroid hormones and undergoes ligand-dependent conformational changes, enabling receptor activation and co-regulator recruitment	[32,36,37,38]
C-Terminal F Domain	Structurally variable region	Function not fully understood; may modulate receptor stability, co-regulator interactions, or transcriptional specificity	[30,39,40,41]

The expression of genes in response to hormonal signals largely depends on the receptors’ structural features, particularly the DNA-binding domain (DBD), the ligand-binding domain (LBD), and their dimerization capabilities. The key structural features and the actions they encompass are shown in Table 2.

The exemplary androgen receptor (UniProt: P10275) is a 920 amino acid protein with distinct functional domains:

A. Domain Structure:

▯ NTD (amino acids 1–559): Largest domain, containing:
  ÷ Activation function 1 (AF-1)
  ÷ Tau-5 region (critical for transactivation)
  ÷ Polyglutamine (poly-Q) and polyglycine (poly-G) repeats
  ÷ Intrinsically disordered region
▯ DBD (amino acids 559–625):
  ÷ Two zinc finger motifs
  ÷ Recognizes androgen response elements (AREs)
  ÷ Highly conserved across steroid receptors
▯ Hinge Region (amino acids 625–676):
  ÷ Contains nuclear localization signals
  ÷ Flexible linker region

**Figure 4:**
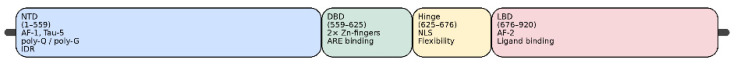
Structural and functional organization of the human androgen receptor (AR). Linear domain architecture of the human androgen receptor showing the intrinsically disordered N-terminal domain (NTD; amino acids 1–559), which contains activation function-1 (AF-1), the Tau-5 transactivation region, and polyglutamine (poly-Q) and polyglycine (poly-G) repeats that collectively mediate ligand-independent transcriptional activity and co-regulator interactions. The centrally located DNA-binding domain (DBD; amino acids 559–625) comprises two zinc finger motifs responsible for androgen response element (ARE) recognition and receptor dimerization. The hinge region (amino acids 625–676) contains nuclear localization signals and provides conformational flexibility linking the DBD to the ligand-binding domain (LBD; amino acids 676–920), which mediates ligand-dependent activation via helix-12/AF-2–dependent co-regulator recruitment. Colors define the extend of spcific domain.

The descriptive representation of the above-described feature is shown in Figure 4.

A hydrophobic cavity within the ligand-binding domain mediates ligand recognition by the androgen receptor and accommodates the steroid nucleus and its substituents. This pocket, with an estimated volume of ~450–500 Å^3^, is formed by key amino acid residues that enable precise positioning of the ligand. Ligands enter the binding site through a channel formed by helices 3, 7, and 11, with evidence for transient peripheral interactions prior to stable engagement of the active site. Binding is stabilized by a network of hydrogen bonds that orient the ligand, complemented by extensive van der Waals contacts between the steroid scaffold and surrounding hydrophobic residues. Among endogenous ligands, dihydrotestosterone exhibits higher affinity and forms a more stable receptor complex than testosterone, reflecting the enhanced potency conferred by 5α-reduction. Critical interactions involve hydrogen bonding between the 3-keto and 17β-hydroxyl groups, and the correct orientation is required; together, these determine agonist efficacy and receptor activation.

Ligand recognition by the androgen receptor is governed by a highly specialized ligand-binding domain (LBD) that integrates pocket geometry, residue composition, and dynamic intermolecular interactions to ensure selective and high-affinity steroid binding. Although structurally optimized to accommodate the conserved steroid nucleus, the AR LBD discriminates between closely related ligands through subtle differences in hydrogen bonding patterns, hydrophobic contacts, and ligand orientation. Endogenous androgens such as testosterone and dihydrotestosterone illustrate how minor chemical modifications can yield pronounced differences in receptor affinity, complex stability, and biological potency. The table below (Table 3) summarizes the key structural and mechanistic features underlying ligand recognition and binding within the AR ligand-binding pocket.

Steroid hormone receptors display a high degree of structural conservation within their ligand-binding domains, yet they are capable of discriminating with remarkable precision among closely related endogenous and synthetic ligands. This selectivity is fundamental to ensuring receptor-specific signaling and avoiding inappropriate cross-activation despite substantial sequence homology between androgen, glucocorticoid, progesterone, and mineralocorticoid receptors. The molecular basis of this specificity arises from subtle but functionally decisive differences in ligand-binding pocket architecture, amino acid composition, and conformational dynamics. The following table (Table 4) summarizes the principal structural determinants that govern ligand selectivity and specificity across steroid hormone receptors, highlighting how variations in pocket geometry, key residue substitutions, receptor flexibility, and auxiliary regulatory mechanisms collectively shape molecular recognition and receptor activation.

Structure–activity relationships (SAR) form the molecular foundation for understanding how anabolic–androgenic steroids (AAS) exert distinct biological effects despite sharing a common steroid backbone. Subtle chemical modifications to the androgen scaffold profoundly influence receptor affinity, agonist potency, pharmacokinetics, and tissue selectivity. Both naturally occurring androgens and synthetic AAS illustrate how alterations at specific carbon positions modulate anabolic versus androgenic activity, oral bioavailability, and receptor selectivity. The table below (Table 5) summarizes the key SAR features of natural and synthetic AAS, highlighting representative compounds, general principles governing androgen receptor activation, and the ongoing development of tissue-selective androgen receptor modulators.

## 3. Sports-Utilized Anabolic–Androgenic Steroids

For over half a century, structural modifications of testosterone have primarily aimed at enhancing its anabolic properties [79,80]. Despite decades of extensive research, the development of an anabolic steroid completely devoid of androgenic effects has not yet been achieved. Although it is recognized that dissociation between anabolic and androgenic activities occurs at the cellular level, the precise molecular mechanisms underlying this phenomenon remain incompletely understood.

**Figure 5:**
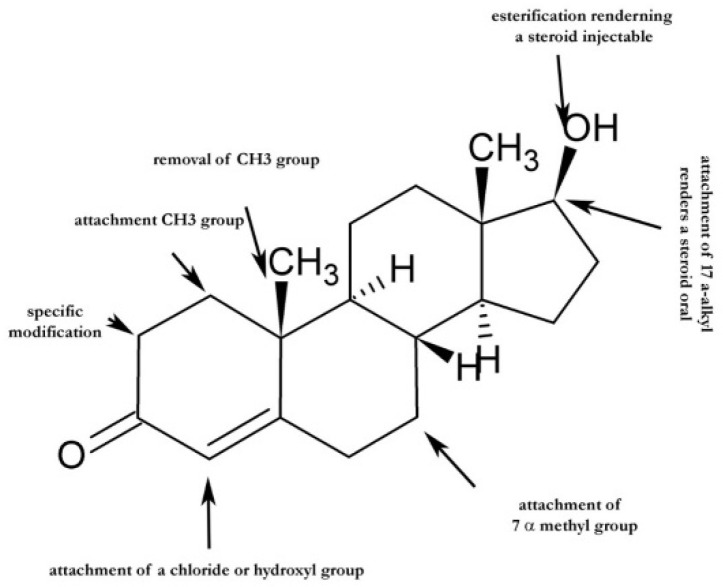
Schematic representation of key structural modification sites on the testosterone steroid backbone used to modulate anabolic and androgenic properties. Highlighted modifications include esterification at C17 to generate injectable formulations, 17α-alkyl substitution to enhance oral bioavailability, removal or addition of methyl groups, introduction of substituents, such as hydroxyl or halogen atoms, on the A-ring, and methyl substitution at C7. Targeted chemical alterations at these positions underlie changes in anabolic potency, androgenic activity, and pharmacokinetic behavior.

As stated in the previous chapter, enhancement of a steroid’s anabolic potency can be achieved through targeted chemical modifications at specific positions within its backbone, as shown in Figure 5.

Anabolic steroids are divided into seven groups: (1) testosterone and C17β-esterified derivatives; (2) 19-Nor derivatives (C19 demethylation); (3) 17α-alkylated steroids (also known as oral anabolic–androgenic steroids); (4) A-ring modified and unsaturated derivatives; (5) heterocyclic A-ring steroids; (6) halogenated steroids; and (7) combined multi-modified steroids.

The respective classes with representative steroids are combined in Table 6.

### 3.1. Class I—Testosterone and C17β-Ester Derivatives

Among all modifiable positions within the steroid nucleus, the C17 carbon represents the single most influential structural determinant governing the biological fate of androgenic steroids. Substitutions at C17 directly regulate androgen receptor (AR) affinity, metabolic stability, route of administration, tissue exposure, and toxicity profiles, thereby exerting a disproportionate impact on both physiological and supraphysiological steroid actions. While modifications at other positions—such as C1 unsaturation, C7 methylation, or C19 demethylation—contribute to anabolic bias and receptor selectivity, alterations at C17 fundamentally define whether a steroid is rapidly inactivated, orally bioavailable, depot-forming, or resistant to hepatic metabolism.

From a biochemical perspective, the C17 position lies at the interface between the D-ring of the steroid nucleus and metabolic enzymes, including hydroxysteroid dehydrogenases, reductases, and hepatic cytochrome P450 systems. Consequently, even subtle chemical changes at this site markedly alter enzymatic accessibility, oxidation–reduction kinetics, and conjugation pathways. These effects translate directly into differences in systemic half-life, plasma concentration–time profiles, and receptor occupancy dynamics.

Importantly, C17 substitutions also shape androgen receptor–ligand interactions. The 17β-hydroxyl group of endogenous androgens participates in critical hydrogen bonding within the AR ligand-binding domain, stabilizing ligand orientation and promoting agonist activity. Chemical modification of this functional group—through oxidation, esterification, alkylation, or replacement—can preserve, attenuate, or profoundly distort receptor activation, thereby influencing anabolic versus androgenic outcomes. As such, C17 chemistry provides a unifying framework that connects structure–activity relationships (SAR), pharmacokinetics, and clinical or illicit use patterns.

Table 7 synthesizes these principles by systematically categorizing androgenic steroids according to the chemical nature of their C17 substitution, highlighting representative compounds and emphasizing the distinct biological and pharmacological properties conferred by each substitution class.

1. 17β-Hydroxyl steroids: Steroids bearing an unmodified 17β-hydroxyl group represent the physiological reference state for androgen signaling. Compounds such as testosterone, dihydrotestosterone (DHT), and 5α-androstanediol exhibit high intrinsic androgen receptor affinity [81] but are rapidly metabolized in the liver, resulting in short systemic half-lives when administered orally. These steroids rely on enzymatic interconversion and local tissue metabolism to fine-tune androgenic signaling [82], reflecting their role in normal endocrine physiology [83].
2. 17-Keto steroids: Oxidation of the 17β-hydroxyl group to a 17-keto moiety yields steroids such as androstenedione and dehydroepiandrosterone (DHEA). This modification reduces direct AR agonist potency [84] while enabling these compounds to function as prohormones, undergoing peripheral conversion to active androgens or estrogens [85]. From a structural standpoint, the 17-keto group alters hydrogen-bonding capacity within the AR ligand-binding pocket, explaining the reduced receptor affinity observed for this class.
3. 17α-Alkylated steroids: When a methyl group (–CH_3_) is attached to the C17 position, this modification (17α-alkylation) significantly improves the steroid’s metabolic stability, allowing it to evade extensive first-pass liver metabolism, which typically hinders the bioavailability of non-alkylated steroids [86,87]. The enhanced anabolic properties associated with these modifications not only improve muscle mass and strength but also confer various physiological benefits, such as increased appetite and energy levels [87]. This substitution sterically shields the C17 position from oxidative metabolism, conferring oral bioavailability and prolonged systemic exposure. Representative compounds—including methyltestosterone, oxandrolone, stanozolol, fluoxymesterone, and methandrostenolone—exhibit high anabolic potency but are associated with dose-dependent hepatotoxicity, reflecting impaired hepatic clearance. Table 7 highlights this class as a paradigm of how chemical stabilization at C17 trades metabolic resistance for increased toxicological burden.
4. 17β-Esterified hydroxyl derivatives: Esterification at the C17 position of steroid molecules facilitates the conversion of testosterone into injectable formulations, thereby significantly enhancing their pharmacokinetic properties and altering the steroid’s solubility and release characteristics [88,89]. Moreover, esterification of the 17β-hydroxyl group with fatty acid chains (e.g., acetate, propionate, enanthate, cypionate, undecanoate) transforms active steroids into lipophilic prodrugs. These esters, exemplified by testosterone enanthate and testosterone cypionate, are biologically inert until hydrolyzed in vivo, enabling depot formation and sustained release following intramuscular administration. Significantly, esterification does not alter intrinsic AR binding geometry; instead, it modulates pharmacokinetics without changing pharmacodynamics, a distinction explicitly emphasized in Table 7.
5. 17-Ether derivatives: Less commonly encountered, 17-ether substitutions replace the hydroxyl proton with an alkyl ether, generating compounds such as testosterone 17-methyl ether. These modifications reduce polarity and metabolic susceptibility while preserving partial receptor activity [90,91,92]. Although historically explored, ether derivatives remain of limited clinical relevance, serving primarily as experimental probes of steroid metabolism.
6. 17-Hydrogen (unsubstituted) steroids: Steroids lacking functional substitution at C17 (e.g., androstane derivatives) exhibit minimal androgenic activity and primarily serve as structural backbones rather than bioactive hormones [64,93,94]. Their inclusion in Table 7 underscores the necessity of appropriate C17 functionalization for meaningful AR engagement.
7. Bulky or heterocyclic C17 substituents: The incorporation of bulky or heterocyclic moieties at C17, as seen in compounds such as danazol and modified nandrolone derivatives, introduces steric and electronic features that can partially dissociate anabolic and androgenic effects. These substitutions often disrupt classical receptor interactions, yielding atypical signaling profiles and, in some cases, partial agonism or antagonism [95,96].
8. 17β-Alkyl (non-ester) substitutions: In contrast to 17α-alkylation, 17β-alkyl substitutions retain the stereochemistry favorable for receptor engagement while modestly enhancing metabolic stability. Certain nandrolone derivatives exemplify this category, highlighting how stereochemical orientation at C17 critically influences biological outcomes.
9. Polar synthetic substituents: Finally, Table 7 includes experimental polar substituents, such as carboxamide-containing side chains, designed to explore novel AR interactions [97]. These compounds underscore ongoing efforts to engineer selective androgen receptor ligands by exploiting C17 chemistry beyond classical anabolic steroid design [98].

Collectively, Table 7 establishes C17 substitution as a central organizing axis for understanding androgenic steroid diversity. By linking chemical functionality to receptor interaction, metabolic fate, and clinical or non-medical use patterns, the table provides a mechanistic bridge between structural chemistry and physiological impact.

Due to its comparatively lower androgenic profile, nandrolone phenylpropionate has historically been reported in female athletic misuse contexts; however, controlled safety data in women remain limited.

#### 3.1.1. 19-Nor Derivatives and Anabolic Bias

Testosterone and its esters (the first group) exert their effects primarily through the androgen receptor, leading to increased protein synthesis, muscle growth, and erythropoiesis. The pharmacokinetics of these esters allow sustained release from the bloodstream following intramuscular injection, thereby providing a more stable hormonal environment than unmodified testosterone [99].

The second group includes compounds such as Nandrolone and trenbolone, which are structurally related to testosterone but modified to enhance anabolic properties while minimizing androgenic side effects. Anabolic steroids in this category tend to have a greater anabolic-to-androgenic ratio, making them more selectively effective for muscle growth with fewer androgenic effects. Nandrolone, for example, has a reduced androgenic property relative to its anabolic activity, making it favorable for specific therapeutic applications [100,101].

17α-Alkylation (third group) refers to the modification at the 17α carbon of the steroid molecule, which improves its oral bioavailability by making it resistant to hepatic metabolism [101,102]. Examples of this group include methyltestosterone, Oxandrolone, and stanozolol.

As outlined above, each group has its unique pharmacokinetic properties, mechanisms of action, and therapeutic applications. While they can provide significant benefits in medical contexts, the potential for misuse, particularly in athletic performance enhancement, raises important health concerns that warrant increased awareness and regulation. Understanding these categories is crucial for healthcare providers, athletes, and policymakers to navigate the complexities of anabolic steroid use and its implications on health.

#### 3.1.2. Heterocyclic and Halogenated Steroids

Ibrahim-Ouali and Dumur [103] provided a comprehensive review of contemporary synthetic strategies for the generation of heterocycle-containing steroid derivatives, highlighting how incorporation of heterocyclic moieties into the steroid scaffold can modulate physicochemical properties, receptor interactions, and biological activity. Such structural modifications may yield compounds with altered pharmacological profiles relative to classical steroids. In this context, Amr and Abdulla [104] reported the synthesis of cyanopyrane-fused steroidal derivatives exhibiting pronounced anti-inflammatory activity in experimental models, with efficacy comparable to selected glucocorticoids, suggesting that heterocycle fusion may represent a viable approach for developing novel steroid-based anti-inflammatory agents with potentially improved safety profiles.

Halogenated steroids are notable for their significant biological activities and have been synthesized for various pharmacological applications. For example, Fattorusso et al. [105] reported the isolation of polychlorinated androstanes from Cliona nigricans, marking a novel entry into the world of halogenated steroids with potential cytotoxic effects against tumor cells. These findings suggest that halogenation can enhance the biological profile of steroids.

The incorporation of halogen atoms can modify the interaction of the steroid with biological targets, potentially altering its pharmacodynamic and pharmacokinetic properties. Wang et al. [106] emphasized the importance of halogenation in steroid modifications for anticancer applications, indicating a structure–activity relationship that merits further investigation. Studies showed that the introduction of halogens into steroidal frameworks can enhance binding affinity for androgen receptors while reducing side effects.

## 4. Sports Employed Anabolic–Androgenic Steroids

### 4.1. Class I—Testosterone and C17β-Esterified Derivatives

#### 4.1.1. Testosterone Propionate

**Figure 6:**
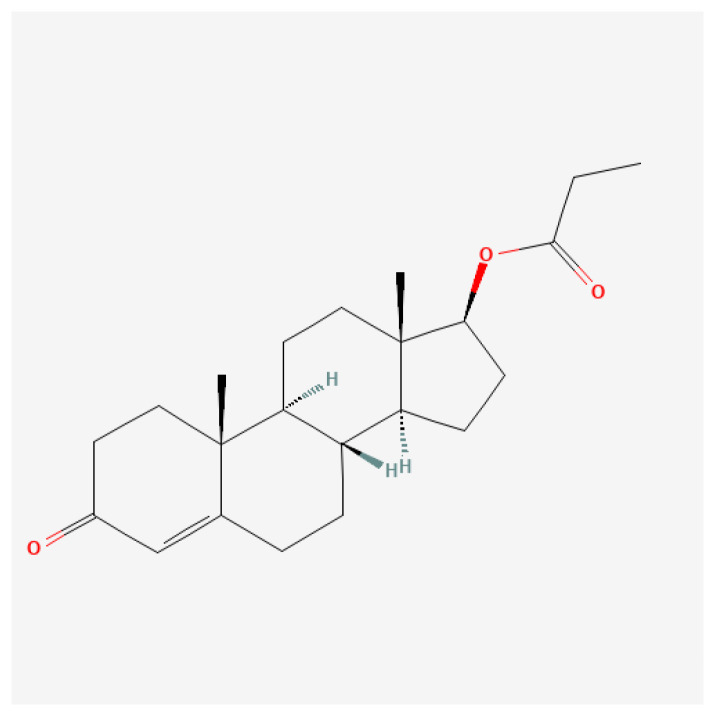
Molecular structure of testosterone propionate, a short-chain ester of testosterone in which the 17β-hydroxyl group is esterified with propionic acid. This short ester chain confers lower lipophilicity and more rapid hydrolysis after intramuscular administration, resulting in a shorter duration of action and faster onset of androgenic effects compared with longer-chain testosterone esters.

Short-chain ester (Figure 6): The pharmacokinetics of testosterone propionate reveal that it is rapidly absorbed after intramuscular injection, leading to swift increases in serum testosterone levels. Studies indicate that TP typically reaches peak serum concentrations within 24–36 h after administration and has a shorter half-life of approximately 0.8–2 days, necessitating more frequent dosing than long-acting esters [107,108]. The implications of this pharmacokinetic profile are significant for both clinical therapies, where precise dosing is critical, and for athletics, where timing of administration can affect performance outcomes. A paper from 1943 stated that 25 mg of testosterone propionate administered three times per week may be used for curing angina pectoralis [109]. A recent study on the physiological response of testosterone propionate revealed the following: (1) propionate has a detrimental role in exercise-induced augmented capillarization (capillarization is the formation and development of a network of capillaries to a part of the body); and (2) it causes mild myocyte hypertrophy of the heart muscle (increase in the volume of an organ or tissue due to the enlargement of its component cells) [110]. In the realm of performance enhancement, testosterone propionate is favored by some athletes for its ability to promote lean muscle mass and strength gains without significant water retention, a common issue with longer-acting steroids [111]. However, it is important to note that the non-medical use of testosterone propionate and other anabolic steroids is often associated with considerable health risks, including adverse effects on cardiovascular health and potential liver damage if misused [112,113]. While testosterone propionate is generally well-tolerated when used appropriately, potential side effects can arise. Common adverse effects include injection site reactions, changes in libido, and mood alterations such as heightened aggression or anxiety [114,115]. Long-term use of testosterone propionate, particularly in supra-therapeutic doses for bodybuilding or athletic purposes, has been linked to more severe complications, including cardiovascular issues and alterations in lipid profiles, increasing the risk for atherosclerosis [111,116].

#### 4.1.2. Testosterone Enanthate

**Figure 7:**
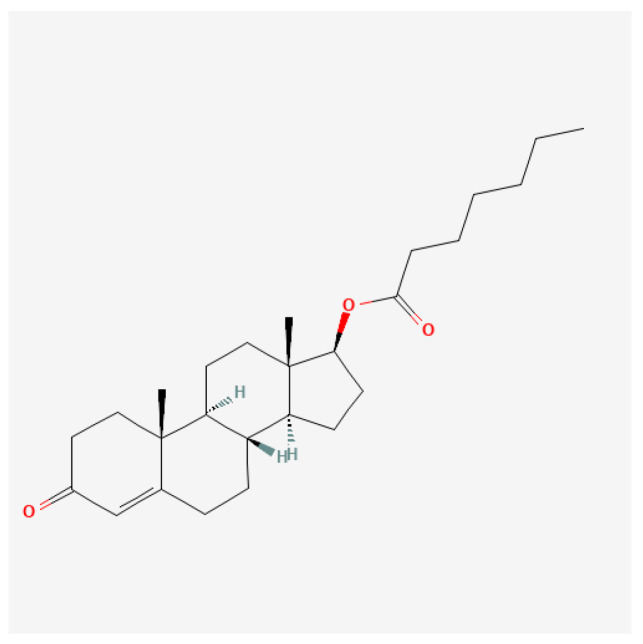
Molecular structure of testosterone enanthate, a long-chain fatty acid ester of testosterone formed by esterification of the 17β-hydroxyl group with enanthic (heptanoic) acid. This modification increases lipophilicity and slows hydrolysis after intramuscular injection, thereby prolonging systemic availability and sustaining androgenic activity. Testosterone enanthate is widely used in clinical practice and research as a long-acting testosterone formulation.

Medium-chain ester (Figure 7): The pharmacokinetic profile of testosterone enanthate indicates that it is well-absorbed when administered intramuscularly. Studies have shown that a single injection of testosterone enanthate results in a significant increase in serum testosterone levels. For instance, a study involving orchidectomized cynomolgus monkeys demonstrated that after a single intramuscular injection, serum testosterone levels peaked and remained elevated for a period, which is crucial for therapeutic efficacy [89]. Furthermore, differing administration routes and dosages significantly affect total testosterone concentrations and the suppression of gonadotropic hormones like luteinizing hormone (LH) [117]. Testosterone enanthate is primarily used to treat male hypogonadism, a condition characterized by low serum testosterone levels. Clinical trials have demonstrated that testosterone therapy can improve various health parameters, including mood, muscle mass, bone density, and overall quality of life [118,119]. In a study involving men with congestive heart failure, testosterone enanthate treatment resulted in better cardiovascular status and functional capacity, underscoring its potential benefits beyond endocrine disorders [118]. Some studies also showed that administering 100 mg/week for 18 months resulted in a decrease in total fat tissue of approximately 14% [120]. Another study, in which the dose of testosterone enanthate was 3 mg/kg body mass/week for 12 weeks, reported a 25% increase in protein synthesis, along with a 20% increase in body mass relative to the control group [121].

#### 4.1.3. Testosterone Cypionate

**Figure 8:**
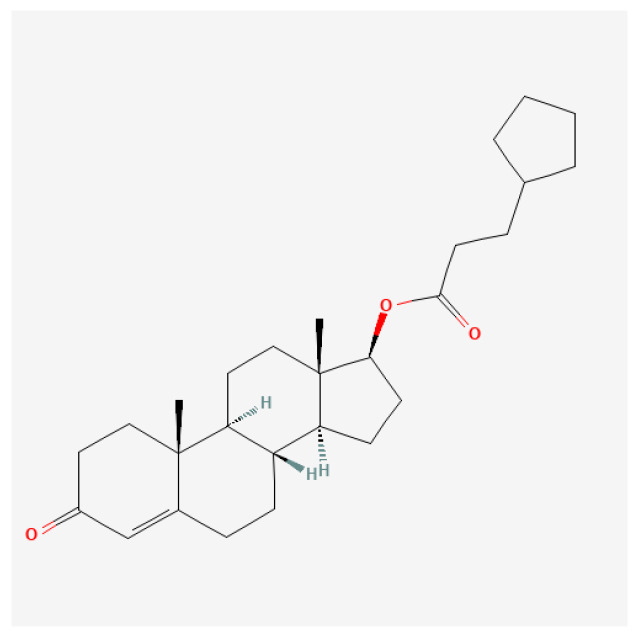
Molecular structure of testosterone cypionate, a synthetic ester of testosterone in which the 17β-hydroxyl group is esterified with cyclopentylpropionic acid. Esterification increases the compound’s lipophilicity and prolongs its release and biological half-life following intramuscular administration, making testosterone cypionate a long-acting androgen widely used in clinical and experimental settings.

Cyclopentylpropionate ester (Figure 8): In therapeutic settings, it is typically administered by intramuscular injection. The most common therapeutic dosage for males with testosterone deficiency is approximately 200–400 mg every 2–4 weeks [122,123,124]. Studies have indicated that supraphysiological doses can markedly enhance lean body mass and muscular strength [123,125]. However, these high doses are also associated with significant health risks, including cardiovascular complications and psychological effects such as increased aggression and mood swings [126,127]. The risk of erythrocytosis increases substantially with higher doses. Regular monitoring of hematocrit levels is crucial in patients receiving supraphysiological doses to mitigate the risks of thromboembolic events [123,128,129].

### 4.2. Class II—19-Nor Derivatives (C19 Demethylation)

#### 4.2.1. Nandrolone Phenylpropionate

**Figure 9:**
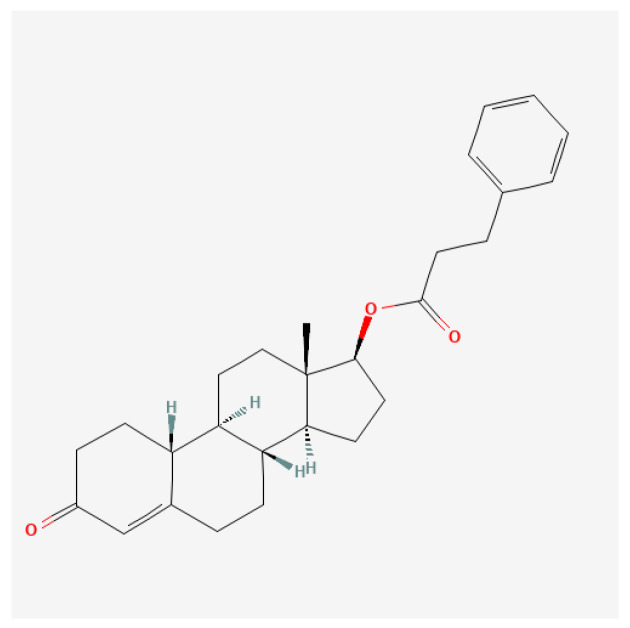
Molecular structure of nandrolone phenylpropionate, a synthetic ester of 19-nortestosterone (Nandrolone) in which the 17β-hydroxyl group is esterified with phenylpropionic acid. The phenylpropionate ester increases lipophilicity and prolongs release following intramuscular administration compared with non-esterified Nandrolone, resulting in intermediate-acting anabolic–androgenic effects.

Nandrolone phenylpropionate (NPP; Figure 9) is a synthetic anabolic–androgenic steroid (AAS) derived from Nandrolone, characterized by a phenylpropionate ester. This esterified form has a relatively short half-life compared to other nandrolone esters, affording a more rapid plasma concentration peak while still facilitating significant anabolic effects. First synthesized in the 1950s, NPP has been utilized therapeutically in clinical settings for conditions such as muscle wasting and osteoporosis but is also frequently associated with non-medical use in athletics for performance enhancement [112].

Nandrolone phenpropionate exhibits a pharmacokinetic profile that allows rapid absorption and clearance. Following intramuscular injection, peak plasma levels are typically reached within 24–48 h. The half-life of NPP is generally 4.5–6 days, necessitating more frequent administration than longer esters such as nandrolone decanoate [130]. This rapid metabolism and clearance may appeal to athletes who desire short bursts of performance enhancement without long-lasting drug traces in their systems. It may exert a more substantial anabolic effect in women than in men [131].

Nandrolone phenpropionate is a steroid often used by women [132]. Experimental and observational reports have described marked strength gains at relatively low daily doses; however, these findings are heterogeneous and context-dependent [71].

#### 4.2.2. Nortestosterone Decanoate

**Figure 10:**
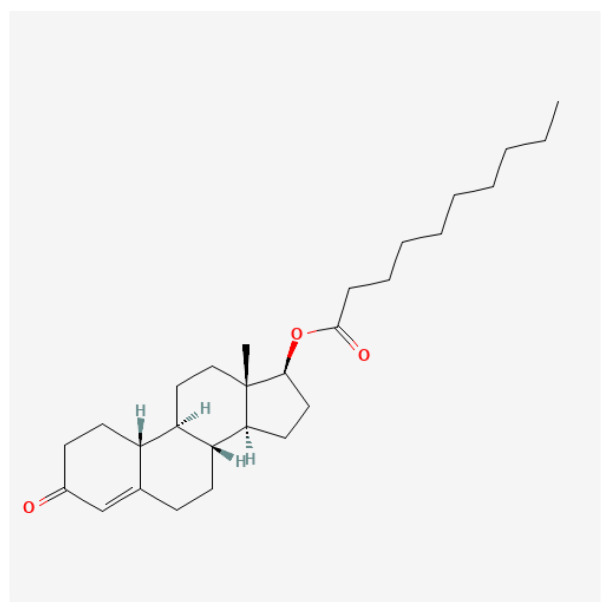
Nandrolone decanoate is a long-acting anabolic–androgenic steroid ester derived from 19-nortestosterone. The molecule consists of the nandrolone steroid nucleus with a decanoate (decanoic acid) ester linked to the 17β-hydroxyl group, which increases lipophilicity and prolongs release after intramuscular administration. Oxygen atoms involved in the ketone and ester functional groups are highlighted, and the stereochemistry of the steroid rings is indicated by wedge and dashed bonds.

Nortestosterone decanoate (Figure 10) is typically administered via intramuscular injection, where its esterified form allows for a sustained release into the bloodstream. The pharmacokinetic profile indicates a peak plasma concentration within a few days post-injection, with significant effects lasting up to several days due to the ester’s long carbon chain [133,134]. This allows for less frequent dosing compared to shorter-acting agents, improving patient compliance in therapeutic settings.

After injection, it is slowly released from muscle tissue, and after 6 days, only 50% of the injected amount will appear in the bloodstream [135]. It is quite a potent steroid and has been shown to improve strength among HIV patients [136], where an increase in body mass and physical strength was observed after 12 weeks of steroid administration. A “sport” effective dose in males is 200–600 mg/week, and the effective dose in women is 50–100 mg/week [137].

Potential adverse effects include (1) endocrine dysfunction: chronic use may lead to decreased natural testosterone production, resulting in secondary hypogonadism [138]; (2) cardiovascular complications: the anabolic effects of ND may be accompanied by shifts in lipid profiles and elevated blood pressure, raising concerns about cardiovascular health; and (3) psychological effects: users may experience mood alterations, aggression, or other psychological issues due to the influence of androgens on the central nervous system [138].

#### 4.2.3. Norbolethone

**Figure 11:**
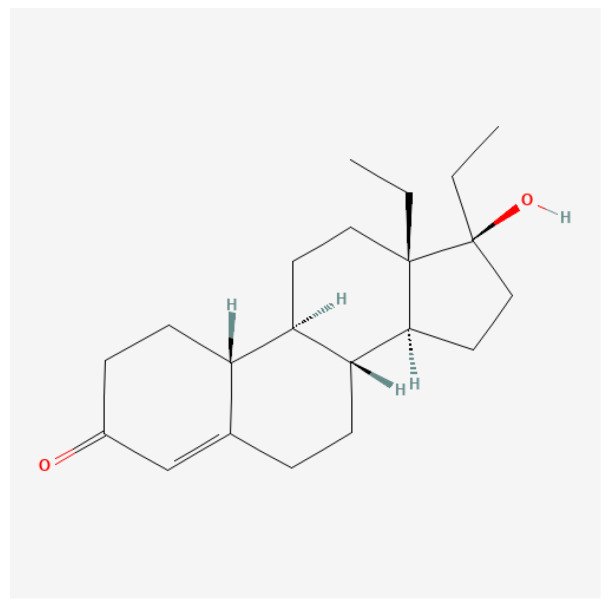
Molecular structure of Norbolethone, a synthetic anabolic–androgenic steroid derived from 19-nortestosterone and characterized by the absence of the C19 methyl group and the presence of a 17β-hydroxyl group. These structural features enhance anabolic potency relative to androgenic activity and confer resistance to rapid metabolic inactivation. Norbolethone has been studied primarily in experimental and anti-doping contexts rather than for therapeutic use.

Norbolethone (13-ethyl-17-hydroxy-18,19-dinor-17α-pregn-4-en-3-one; Figure 11) is a 19-nor anabolic steroid first synthesized in the 1960s. While it was developed initially for possible medical use, it never reached the commercial market. However, Norbolethone has attracted attention in both clinical research and doping control due to its anabolic properties, particularly in athletics and bodybuilding contexts [63,64]. This synthetic steroid has been detected in doping tests, indicating its illicit use among athletes [139].

Norbolethone exerts its effects primarily through androgen receptor activation, leading to increased protein synthesis and muscle growth. This mechanism is consistent with the actions of other anabolic steroids [140,141]. Studies indicate that Norbolethone can enhance nitrogen retention and may have protective effects against certain types of toxicities, such as those produced by digitalis compounds [140]. This anabolic steroid may also influence male reproductive physiology and certain metabolic pathways, although data specific to these effects are limited.

Detection of Norbolethone poses a significant challenge due to its structural similarities with other anabolic agents and its limited prior documentation in doping control. Advances in liquid chromatography–tandem mass spectrometry have enabled the identification of this compound in urine samples from athletes, indicating its clandestine use [139,142]. The lack of prior evidence among doping violators underscores the need for ongoing surveillance in sports medicine.

The main advantage of this steroid is its lack of conversion to estrogen [143] and its very low androgenic activity [144]. One study claimed it is toxic [139], but no clinical studies have supported this claim.

### 4.3. Class III—17α-Alkylated Steroids (Oral AAS)

#### 4.3.1. Methandrostenolone

**Figure 12:**
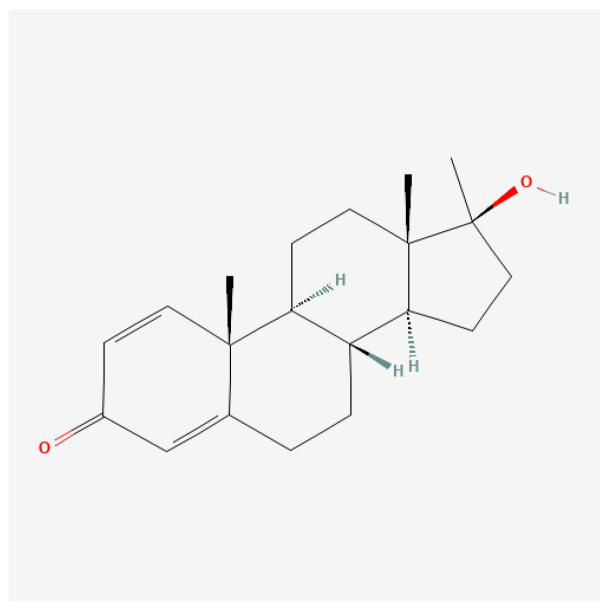
Chemical structure of methandrostenolone (17α-methyl-1-dehydrotestosterone). The figure illustrates the molecular structure of methandrostenolone, a synthetic anabolic–androgenic steroid derived from testosterone and characterized by two key structural modifications: 17α-methylation and Δ^1^ unsaturation (C1–C2 double bond) within the A-ring. The steroid retains the classical tetracyclic cyclopentanoperhydrophenanthrene backbone, with a 3-keto group on the A-ring and a 17β-hydroxyl group on the D ring, both of which are essential for androgen receptor (AR) binding. The 17α-methyl substituent confers resistance to first-pass hepatic metabolism and enables oral bioavailability, whereas Δ^1^ unsaturation alters the electronic distribution of the A-ring, contributing to an increased anabolic-to-androgenic activity ratio. Stereochemistry is indicated by wedge and dashed bonds, highlighting the preserved three-dimensional configuration of the steroid nucleus.

Widely recognized as Dianabol (Dbol), methandrostenolone (Figure 12) is a synthetic anabolic steroid first synthesized in the 1950s. It gained immense popularity among athletes and bodybuilders due to its potent muscle-building properties and performance-enhancing effects. Initially developed for therapeutic applications, methandrostenolone has transitioned into one of the most misused anabolic steroids in sports, leading to significant health concerns and legal implications [145].

As an oral anabolic steroid, methandrostenolone is rapidly absorbed into the bloodstream. Peak plasma levels are reached approximately 2 to 4 h after ingestion, with a half-life of about 3 to 6 h [146]. This pharmacokinetic profile necessitates frequent dosing to maintain physiological effects, often leading to higher cumulative dosages in users compared to injectable steroids.

In the realm of sports, methandrostenolone has become a staple among bodybuilders and athletes, primarily for its capacity to enhance strength and accelerate muscle growth during bulking phases of training. Studies have documented its efficacy in promoting significant increases in muscle mass, strength, and recovery time [146]. Reported non-medical use in athletic populations has included cumulative annual exposures of several grams, although dosing practices vary widely and are poorly standardized [147,148]. Studies have shown that anabolic steroids effectively promote the growth of skeletal muscle fibers, which may enhance athletic performance [149,150]. The steroid has been associated with alterations in muscle cell histochemistry, augmenting the size and number of muscle fibers [149]. However, its efficacy can be influenced by concurrent training regimens, with research indicating that while methandrostenolone can improve muscle metrics, training alone has significant benefits for muscle composition and performance [146,149].

Despite its efficacy in enhancing muscle mass, methandrostenolone presents numerous potential side effects that warrant caution among users. These include androgenic effects such as acne, hair loss, and gynecomastia, as well as possible liver toxicity evidenced by elevated liver enzymes in continuous users [145,146,151]. Furthermore, psychological effects such as aggression, anxiety, and mood swings have been documented [152,153].

In girls and women, the use of methandrostenolone can result in virilization effects, encompassing deepening of the voice and increased body hair [150,153]. Chronic use can also lead to reproductive health problems, particularly in male users, due to testosterone suppression and subsequent infertility risks [149,150].

#### 4.3.2. Oxandrolone

**Figure 13:**
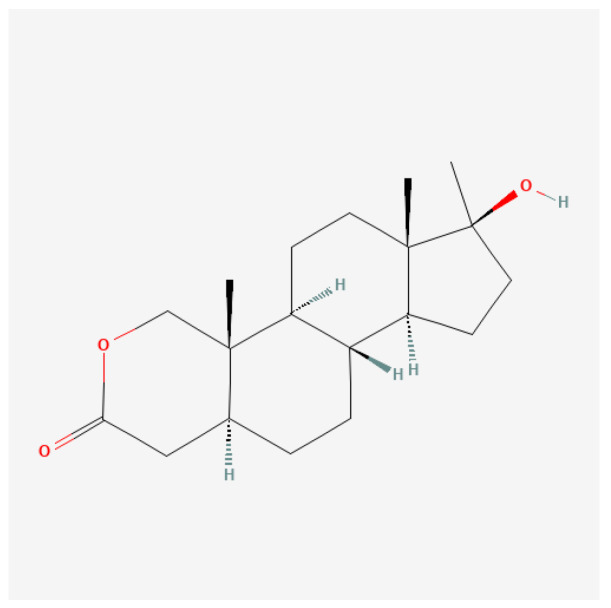
Oxandrolone is a synthetic anabolic–androgenic steroid derived from dihydrotestosterone. The molecule consists of a modified steroid nucleus containing a 17α-methyl group and an oxygen atom incorporated into the A-ring (2-oxa substitution), structural features that enhance oral bioavailability and metabolic stability. Oxygen atoms involved in the ketone and hydroxyl functional groups are highlighted, and the stereochemistry of the steroid rings is indicated by wedge and dashed bonds.

Oxandrolone (Figure 13), commonly known as Anavar, is a synthetic anabolic–androgenic steroid (AAS) derived from dihydrotestosterone (DHT). Compared to testosterone, its androgenic activity is quite low, and it is not hepatotoxic [154,155], making it particularly attractive for various therapeutic applications. Initially introduced in the 1960s, Oxandrolone has remained relevant in both clinical and athletic contexts due to its efficacy in promoting weight gain and muscle mass without the pronounced side effects associated with many other anabolic steroids [156]. Its metabolites can be detected in anti-doping tests for up to three weeks after cessation of drug administration [137].

Oxandrolone is administered orally and exhibits relatively high bioavailability. Following administration, peak plasma concentrations are typically observed within 2 to 4 h, with a half-life ranging from approximately 9 to 10 h. This profile allows for flexible dosing schedules, making it suitable for both therapeutic and non-medical users [157,158]. Oxandrolone is known for its ability to maintain muscle mass, promote protein synthesis, and facilitate fat loss, making it popular among bodybuilders and athletes [159].

In sports, Oxandrolone is frequently misused by athletes and bodybuilders seeking to improve strength, muscle mass, and performance. Its favorable anabolic-to-androgenic ratio makes it appealing for enhancing physical performance without significantly increasing the risk of androgenic side effects such as hair loss or acne [157,159].

Oxandrolone is associated with several potential side effects, including (1) liver toxicity: as an oral anabolic steroid, Oxandrolone can induce liver strain, necessitating regular monitoring of liver function in long-term users [116,160]; (2) hormonal imbalance: prolonged use can suppress natural testosterone production, potentially leading to secondary hypogonadism and fertility issues [159,161]; cardiovascular effects: Oxandrolone may alter lipid profiles, increasing the risk of cardiovascular diseases through reduced high-density lipoprotein (HDL) cholesterol levels [157,162]; and (3) psychological changes: users might experience mood swings or aggression, common with anabolic steroid use, although Oxandrolone has a lower incidence of such effects compared to more potent AAS [163].

#### 4.3.3. Stanozolol

**Figure 14:**
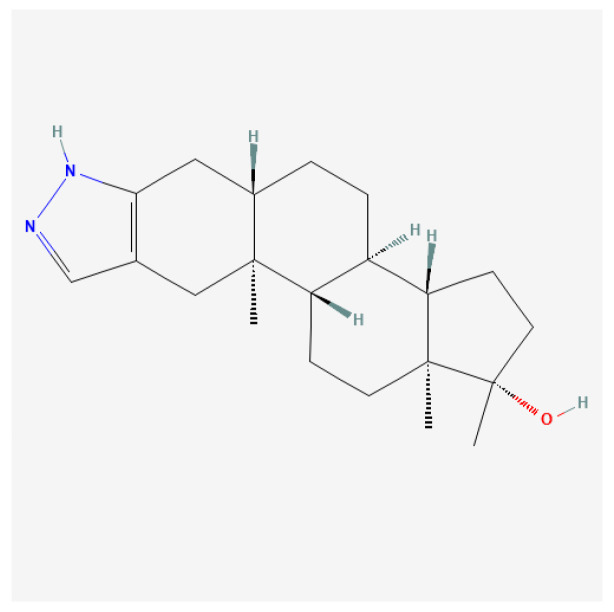
Chemical structure of stanozolol, a synthetic anabolic–androgenic steroid derived from dihydrotestosterone. The molecule features the characteristic tetracyclic steroid nucleus fused to a pyrazole ring at the A-ring, a structural modification that distinguishes stanozolol from testosterone derivatives and contributes to its pharmacological profile. A hydroxyl group is present at the C17 position, with the oxygen atom highlighted, while nitrogen atoms within the heterocyclic pyrazole ring are shown in blue. The three-dimensional stereochemistry of the fused rings and substituents is indicated by solid wedges and dashed bonds.

Stanozolol (Figure 14), commonly known by its brand name Anavar, is a synthetic anabolic steroid derived from dihydrotestosterone (DHT). Initially developed in the 1960s, stanozolol possesses significant anabolic properties and is characterized by relatively low androgenic effects; therefore, it is readily used by women [132].

Certain anabolic–androgenic steroids, such as stanozolol, combine multiple structural modifications; in such cases, classification is based on the dominant determinant of pharmacokinetic behavior, with additional modifications discussed separately for their mechanistic relevance.

Stanozolol binds to androgen receptors in skeletal muscle, promoting enhanced protein synthesis and muscle growth while also having beneficial effects on endurance and recovery [164,165]. Unlike many other anabolic steroids, stanozolol is notable for its ability to maintain muscle mass while minimizing fat accumulation, making it particularly appealing to those engaged in resistance training or bodybuilding.

Stanozolol is typically taken orally, leading to rapid absorption. Peak plasma concentration occurs within 1 to 2 h after administration, with a half-life of approximately 9 h [166,167]. This necessitates multiple doses throughout the day to maintain optimal steroid levels in the body. It is classified as a 17-alpha-alkylated steroid, which provides oral bioavailability but also includes a risk for liver toxicity compared to injectable forms [167].

Stanozolol has been employed in clinical settings for various conditions, particularly in the following areas: (1) muscle wasting disorders: stanozolol is indicated for use in patients experiencing severe weight loss due to chronic illnesses such as cancer or HIV/AIDS, facilitating an increase in lean body mass and improving general energy levels [167,168]; (2) anemia: it is also prescribed to enhance erythropoiesis, helping increase red blood cell production, particularly in individuals with chronic anemia due to renal disease [167]; and (3) osteoporosis: due to its anabolic effects on bone density, stanozolol has potential applications in the treatment of osteoporosis, particularly in older adults at risk of fractures [165].

The abuse of stanozolol is linked to numerous side effects, some of which can have long-term consequences: (1) liver toxicity: stanozolol can lead to liver damage, particularly at high doses or with prolonged use. Liver function should be monitored in patients receiving this medication [166,167]; (2) hormonal disruption: extended use can suppress natural testosterone levels, leading to symptoms of hypogonadism, such as reduced libido, testicular atrophy, and infertility [165,169]; (3) cardiovascular issues: stanozolol can negatively affect lipid profiles by decreasing HDL cholesterol and increasing LDL cholesterol, raising the risk of cardiovascular diseases [87,167]; and (4) psychological effects: users may experience psychological side effects, including mood swings, aggression, and increased anxiety. The extent of these effects can vary significantly between individuals [2,87].

### 4.4. Class IV—A-Ring Modified/Unsaturated Derivatives

#### Boldenone (Δ^1^)

**Figure 15:**
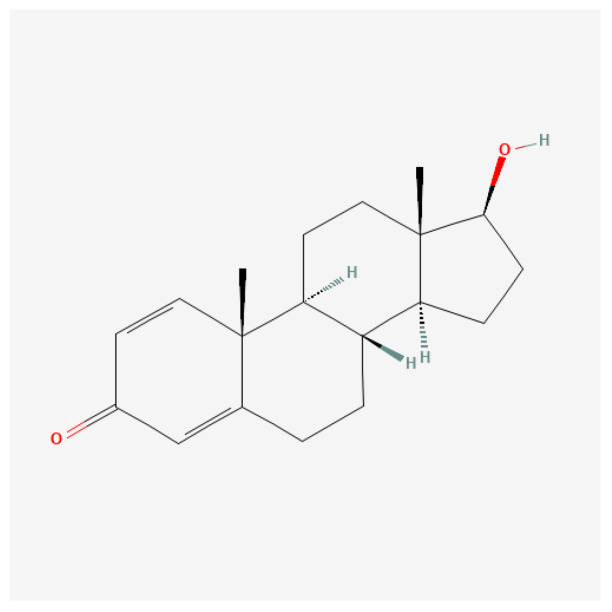
Chemical structure of boldenone (Δ^1^-testosterone). The figure depicts the molecular structure of boldenone, an anabolic–androgenic steroid derived from testosterone and characterized by the presence of an additional double bond between C1 and C2 (Δ^1^ unsaturation) in the A-ring. The steroid nucleus retains the classical tetracyclic cyclopentanoperhydrophenanthrene scaffold, with a 3-keto group on the A-ring and a 17β-hydroxyl group on the D ring, both of which are critical for androgen receptor (AR) binding. Introduction of the Δ^1^ double bond alters the electronic distribution and planarity of the A-ring, reducing susceptibility to aromatization and shifting the anabolic-to-androgenic activity ratio relative to testosterone. Hydrogen atoms and stereochemistry are indicated by dashed and wedge bonds, highlighting the preserved three-dimensional configuration of the steroid backbone.

Boldenone (Figure 15) acts predominantly by enhancing protein synthesis, contributing to muscle hypertrophy and improved recovery times. Its anabolic properties arise due to its structural modifications from testosterone, granting it unique biochemical interactions and a more prolonged release profile when used as an undecylenate ester. The anabolic effect is often pronounced in controlled-dosing scenarios, where growth parameters are meticulously tracked in livestock and athletic populations [170,171,172].

Recent studies have highlighted notable adverse effects of boldenone administration on various organs, specifically the liver and kidneys. In experimental models using rabbits, injections of boldenone have been shown to alter renal and hepatic morphology and function. For instance, injections led to deterioration of the glomerular structure and compromised kidney function, as indicated by increased serum creatinine and altered serum globulin levels [170,171,173]. Furthermore, the hormone exhibits potential hepatotoxicity, leading to structural and functional changes in liver tissue following administration [172,173].

In another study, the histopathological effects of boldenone were characterized by marked toxic changes in the liver and kidneys, including cellular apoptosis and inflammatory responses [173,174]. This emphasizes the importance of monitoring hepatic and renal function in individuals undergoing treatment with this anabolic steroid, particularly due to the elevation of liver enzymes and signs of hepatic distress evident in multiple instances following boldenone exposure [172,173,174].

### 4.5. Class V—Heterocyclic A-Ring Steroids

#### Danazol

**Figure 16:**
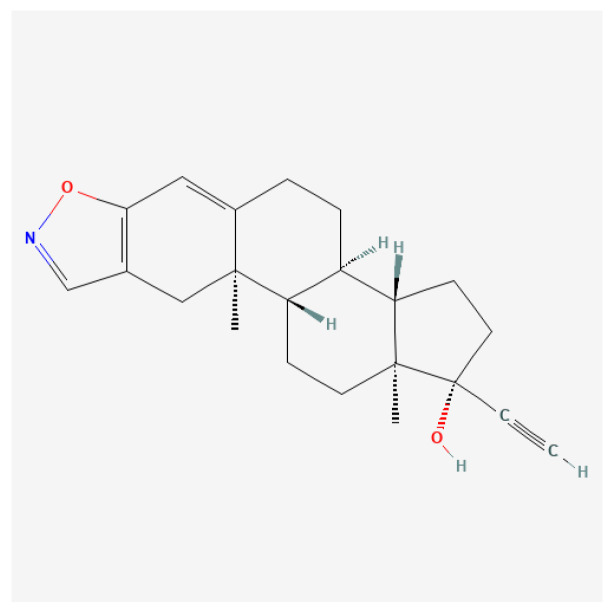
Chemical structure of danazol. The figure depicts the molecular structure of danazol, a synthetic steroid derivative characterized by fusion of an isoxazole heterocycle to the A-ring and the presence of an ethinyl (–C≡CH) substituent at the C17 position. The compound retains the tetracyclic steroid backbone but exhibits substantial modifications to the A-ring electronics and D-ring substituent chemistry relative to testosterone. These structural features alter hydrogen-bonding patterns and steric interactions within the androgen receptor (AR) ligand-binding domain, contributing to danazol’s atypical endocrine profile and partial androgenic activity. Heteroatoms within the isoxazole ring (oxygen and nitrogen) and the 17β-hydroxyl group are highlighted, and stereochemistry is indicated by wedge and dashed bonds.

Danazol (Figure 16) works primarily by suppressing ovarian function, leading to a decrease in the secretion of gonadotropin-releasing hormone (GnRH) and consequently inhibiting luteinizing hormone (LH) and follicle-stimulating hormone (FSH) levels. As a result, danazol induces low, non-cyclic serum estradiol levels, which mitigate the symptoms associated with estrogen-dependent disorders, notably endometriosis and menorrhagia [175,176,177].

The mechanism behind its therapeutic efficacy in endometriosis involves reduced endometrial tissue proliferation due to suppressed estrogen levels, leading to improved symptomatology, including alleviation of pain and reduced menstrual bleeding [176,178,179].

### 4.6. Class VI—Halogenated Steroids

#### Fluoxymesterone (9α-Fluoro)

**Figure 17:**
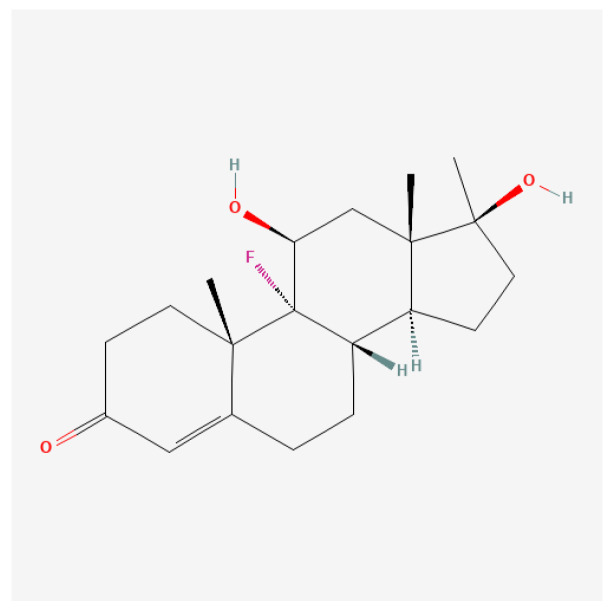
Fluoxymesterone is a synthetic anabolic–androgenic steroid derived from testosterone. The molecule consists of the steroid nucleus bearing a 17α-methyl group, a 9α-fluoro substitution, and additional hydroxyl functionalization, structural features that enhance oral bioavailability and androgen receptor affinity. Oxygen atoms involved in the ketone and hydroxyl functional groups, as well as the fluorine substitution, are highlighted, and the stereochemistry of the steroid rings is indicated by wedge and dashed bonds.

Fluoxymesterone (Figure 17), commonly known by its trade name Halotestin, is a synthetic androgenic anabolic steroid (AAS) derived from testosterone. It is characterized by its potent androgenic activity and moderate anabolic effects, which have made it a subject of interest in various therapeutic contexts, particularly in oncology and endocrine disorder treatments.

Fluoxymesterone exerts its effects primarily by binding androgen receptors (AR) in target tissues. Upon binding to AR, it initiates a sequence of events that lead to the transcription of genes responsible for male sexual development and anabolic processes, such as muscle growth and red blood cell production [180,181]. Unlike some other AAS that primarily emphasize anabolic effects, fluoxymesterone is noted for its significant androgenic properties, making it effective in both treating hypogonadism and managing certain cancer types where anabolic activity is advantageous [180,182].

While fluoxymesterone serves various therapeutic roles, it also carries a spectrum of potential side effects. Commonly reported adverse effects include androgenic symptoms such as acne, hirsutism, voice deepening in women, and potential liver toxicity due to its 17-alpha-alkylated structure [180,183]. Importantly, the effects on lipid profiles and cardiovascular risk factors necessitate vigilant monitoring in treated patients, particularly those with preexisting conditions [180,181].

There is also concern regarding the impact of fluoxymesterone on aggressive behaviors and mood alterations in male and female patients, linked to heightened androgenic activity [182,184]. Clinicians must weigh these risks against the potential benefits, tailoring treatment to minimize harmful effects while maximizing therapeutic outcomes.

Clinical evidence suggests that fluoxymesterone usage may improve body weight and overall physical functioning in these patient populations by counteracting the muscle loss common with these diseases [112].

Fluoxymesterone is associated with several potential side effects: (1) endocrine dysregulation: prolonged use often results in suppression of gonadotropins and endogenous testosterone, potentially leading to infertility and hormonal imbalances [185,186]; (2) liver toxicity: like many oral anabolic steroids, fluoxymesterone is hepatotoxic, with risks of liver damage, particularly at high doses or with long-term use [187]; and (3) cardiovascular effects: alterations in lipid profiles, including reduced high-density lipoprotein (HDL) cholesterol levels, increase the risk of cardiovascular diseases [188].

Psychological Effects: Users can experience mood swings, aggression, and other psychological disturbances, markers commonly associated with steroid abuse [184,189].

## 5. Conclusions

Anabolic–androgenic steroids (AAS) represent a unique pharmacological class in which minor chemical modifications imposed upon a highly conserved steroid scaffold translate into disproportionately large biological effects. Despite decades of clinical use, non-medical misuse, and extensive experimental investigation, the mechanistic basis underlying anabolic–androgenic dissociation has remained conceptually fragmented. The present narrative review integrates advances in steroid structural chemistry, androgen receptor (AR) biology, and intracellular signaling to provide a coherent framework linking molecular structure to receptor-level mechanisms and downstream physiological outcomes.

A central conclusion emerging from this synthesis is that anabolic–androgenic dissociation cannot be explained by receptor binding affinity alone. While the androgen receptor remains the primary molecular mediator of AAS action, ligand-induced differences in receptor conformation, ligand-binding domain (LBD) dynamics, helix-12 positioning, and co-regulator recruitment critically shape transcriptional and non-transcriptional signaling outputs. Subtle alterations in hydrogen-bonding patterns, steric complementarity, and pocket occupancy—particularly within the AR LBD—produce signaling biases that extend beyond classical genomic paradigms. These biases help explain why structurally related steroids can diverge markedly in anabolic potency, androgenic liability, cardiovascular impact, and metabolic risk.

The review further emphasizes that C17 substitution chemistry constitutes a dominant organizing axis in androgen pharmacology. Modifications at the C17 position—whether oxidation to a keto group, esterification of the 17β-hydroxyl moiety, or 17α-alkylation—exert a disproportionate influence on metabolic stability, route of administration, systemic exposure, and toxicological burden. Importantly, these chemical alterations do not merely modulate pharmacokinetics; they also reshape receptor engagement by altering ligand orientation and interaction networks within the AR binding pocket. The distinction between pharmacodynamic neutrality (e.g., 17β-esterification) and pharmacodynamic distortion (e.g., 17α-alkylation) highlights how chemical stabilization can preserve, bias, or impair physiological androgen signaling.

Beyond C17 chemistry, additional structural modifications—including C19 demethylation (19-nor steroids), A-ring unsaturation, heterocyclic fusion, and halogenation—illustrate how multi-site alterations interact non-linearly to generate complex anabolic–androgenic profiles. These interactions underscore the limitations of simple anabolic-to-androgenic ratios as predictive metrics and reinforce the need for receptor-centric, structure-informed interpretations of steroid action. In this context, the historical development of AAS can be viewed not as a linear optimization process, but as an iterative exploration of receptor conformational space constrained by metabolic and toxicological trade-offs.

A further key conclusion is that androgen signaling operates as an integrated, multi-layered network rather than a binary genomic versus non-genomic system. Rapid extranuclear signaling events—mediated through membrane-associated receptors, kinase cascades, calcium fluxes, and cytoskeletal interactions—intersect dynamically with nuclear AR activity. This crosstalk enables context-dependent amplification or attenuation of transcriptional responses, particularly under conditions of mechanical loading, metabolic stress, inflammation, and neuroendocrine adaptation. Such integration is especially relevant in athletic and supraphysiological exposure scenarios, where training-induced signaling pathways may synergize with or exacerbate steroid-driven effects.

From a translational perspective, the review highlights that many adverse outcomes associated with AAS misuse—particularly cardiovascular, hepatic, and neuropsychiatric effects—are not incidental but mechanistically linked to structural features that confer metabolic resistance and prolonged receptor activation. The same chemical modifications that enhance oral bioavailability or depot formation also increase the likelihood of sustained receptor occupancy, off-target interactions, and disrupted physiological feedback loops. These mechanistic insights reinforce the notion that anabolic enhancement and long-term safety are intrinsically coupled through shared molecular determinants.

Importantly, this synthesis also clarifies why the long-standing goal of achieving complete anabolic–androgenic dissociation has remained elusive. Because anabolic and androgenic effects arise from overlapping receptor mechanisms, shared co-regulator pools, and tissue-specific metabolic environments, absolute separation is unlikely within the constraints of steroidal scaffolds. Nonetheless, partial dissociation—manifested as tissue selectivity or signaling bias—remains achievable and continues to inform the rational design of next-generation androgen receptor modulators.

The framework presented here carries implications beyond sports medicine and anti-doping science. By linking ligand structure to receptor dynamics and signaling topology, it provides a conceptual basis for developing safer therapeutic androgens, interpreting heterogeneous clinical outcomes, and refining risk assessment strategies for supraphysiological androgen exposure. Moreover, integrating structural biology with applied physiology underscores the value of cross-disciplinary approaches in resolving complex endocrine paradoxes.

In conclusion, anabolic–androgenic steroids should be understood not as uniformly acting agents, but as structurally encoded signaling modulators whose biological effects emerge from the interplay between chemical architecture, receptor conformational plasticity, intracellular signaling context, and tissue-specific regulation. Recognizing this complexity moves the field beyond reductionist models and toward a more mechanistically faithful understanding of androgen biology. Future progress will depend on integrative studies combining high-resolution structural analyses, systems-level signaling interrogation, and longitudinal human data to translate molecular insight into predictive and clinically meaningful frameworks.

## 6. Limitations

Despite the comprehensive scope of this narrative review, several limitations should be acknowledged. First, the review does not follow a systematic review or meta-analytic framework. Although a structured literature search strategy was employed to identify relevant experimental, clinical, and structural biology studies, the selection and synthesis of evidence remain inherently subject to selection bias. Consequently, the review emphasizes mechanistic representation and conceptual integration rather than exhaustive quantitative coverage of all available studies or compounds.

Second, the evidence base underlying anabolic–androgenic steroid (AAS) biology is highly heterogeneous, encompassing in vitro receptor assays, animal models, clinical investigations, historical pharmacological studies, and observational reports related to non-medical use in athletic populations. These sources differ substantially in experimental design, dosing paradigms, endpoints, and translational relevance. While this review integrates findings across these domains to elucidate structure–activity relationships and receptor-level mechanisms, direct extrapolation from experimental models to long-term human outcomes—particularly in the context of supraphysiological AAS exposure—should be interpreted with caution.

Third, many mechanistic insights discussed herein, including non-genomic androgen receptor signaling, tissue-specific co-regulator recruitment, and signaling bias induced by synthetic steroid modifications, are derived predominantly from cellular or preclinical studies. Although these mechanisms provide a robust theoretical framework for understanding anabolic–androgenic dissociation, their quantitative contribution to physiological adaptation, performance enhancement, and adverse cardiovascular outcomes in humans remains incompletely defined. Future studies integrating molecular, imaging, and longitudinal clinical data will be required to validate these pathways in vivo.

Fourth, the classification of AAS according to structural features—particularly C17 substitution patterns—offers a mechanistically coherent framework but necessarily simplifies the complexity of steroid pharmacology. Individual compounds often possess multiple concurrent modifications that interact non-linearly to influence receptor affinity, metabolic stability, tissue distribution, and signaling outcomes. Accordingly, the categorizations presented in this review should be viewed as heuristic constructs rather than rigid pharmacological boundaries.

Finally, the discussion of non-medical and athletic use of AAS is constrained by the limited availability of high-quality, controlled human data. Much of the literature on performance enhancement, dosing practices, and long-term health consequences is observational or retrospective and may be influenced by reporting bias, underreporting, or confounding lifestyle factors. As a result, associations described in this context should not be interpreted as causal, and the review does not endorse or promote non-medical AAS use.

Taken together, these limitations reflect the current state of the field rather than deficiencies of the present analysis. By integrating structural biology, receptor signaling, and applied physiology, this review aims to provide a mechanistic framework that can guide hypothesis-driven research, inform clinical interpretation, and support future systematic and translational investigations into anabolic–androgenic steroid biology.

## Figures and Tables

**Table 2:** Key structural features governing nuclear steroid hormone receptor function. The table summarizes critical structural elements of nuclear steroid hormone receptors and their associated functional roles in transcriptional regulation. Highlighted features include the DNA-binding domain (DBD) responsible for recognition of hormone response elements (HREs), the ligand-binding domain (LBD) containing a hydrophobic pocket for steroid accommodation, and helix 12 (H12), which is essential for activation function-2 (AF-2)–dependent co-regulator recruitment. In addition, receptor dimerization, either as homodimers or heterodimers with retinoid X receptor (RXR), is emphasized as a fundamental requirement for effective DNA binding and gene regulation. Together, these structural determinants underpin ligand specificity, receptor activation, and tissue-dependent transcriptional outcomes.

Structural Feature	Domain Involved	Functional Significance	References
Recognition of hormone response elements (HREs)	DNA-Binding Domain (DBD)	Enables specific binding to target gene promoters and transcriptional regulation	[42,43]
Hydrophobic ligand-binding pocket	Ligand-Binding Domain (LBD)	Accommodates steroid ligands and stabilizes receptor–ligand complexes	[38,44,45,46,47]
Helix 12 (H12) regulating AF-2 function	Ligand-Binding Domain (LBD)	Controls ligand-dependent receptor activation and co-regulator recruitment	[38,45,47,48]
Dimerization capability	Full receptor (LBD/DBD interfaces)	Allows formation of homo- or heterodimers (e.g., with RXR), essential for DNA binding and transcriptional control	[43,49,50,51,52]

**Table 3:** Molecular features governing ligand recognition and binding within the androgen receptor ligand-binding domain (LBD). The table summarizes the structural characteristics of the androgen receptor ligand-binding pocket, key residues involved in ligand accommodation, and the principal molecular interactions that stabilize receptor–ligand complexes. It also compares the binding properties of endogenous androgens, highlighting differences in affinity and complex stability between testosterone and dihydrotestosterone, and outlines the mechanistic basis by which hydrogen bonding, hydrophobic contacts, and ligand orientation determine agonist potency and receptor activation.

Category	Structural/Mechanistic Feature	Functional Significance
Ligand-binding pocket characteristics	Hydrophobic cavity within the LBD	Provides a non-polar environment optimized for steroid binding
	Accommodation of steroid nucleus and side chains	Enables binding of endogenous and synthetic androgens
	Key residues: Gln738, Met742, Tyr739, His874, Asn770, Cys942, Thr945	Mediate ligand positioning through hydrogen bonding and hydrophobic interactions
	Pocket volume ~450–500 Å^3^	Restricts ligand size and contributes to receptor selectivity
Binding mechanism	Ligand entry via helices 3, 7, and 11	Defines the primary access pathway to the active site
	Peripheral binding site preceding active site engagement	Facilitates ligand guidance and orientation before stable binding
	Hydrogen bonding network	Ensures correct ligand orientation and receptor activation
	Van der Waals contacts	Stabilize the ligand within the hydrophobic pocket
Natural ligands	Testosterone (T): Kd ~1–5 nM	Functions as a weaker AR agonist
	Dihydrotestosterone (DHT): Kd ~0.1–0.5 nM	Acts as a more potent agonist with higher binding affinity
	Enhanced stability of AR–DHT complex	Contributes to prolonged receptor activation
	5α-reduction of testosterone to DHT	Increases androgenic potency by approximately 2–5-fold
Key molecular interactions	3-keto group hydrogen bonds with Gln711 and Arg752	Critical for anchoring the A-ring of the steroid
	17β-hydroxyl group hydrogen bonds with Asn705 and Thr877	Stabilizes ligand orientation within the pocket
	Steroid nucleus hydrophobic contacts	Promote strong van der Waals stabilization
	A-ring orientation	Determines agonist versus antagonist activity

**Table 4:** Structural determinants of ligand selectivity among steroid hormone receptors. Key structural features governing ligand discrimination across nuclear steroid hormone receptors are summarized, including ligand-binding domain (LBD) sequence identity, pocket geometry, critical residue substitutions, conformational flexibility, and induced-fit mechanisms. Additional factors, such as pre-receptor metabolism and targeted synthetic modification, are highlighted as contributors to receptor-specific activation despite conserved steroid scaffolds.

Aspect	Key Features	Structural/Functional Implications
Receptor selectivity (LBD sequence identity)	AR vs. GR ≈ 55%; AR vs. PR ≈ 53%; AR vs. MR ≈ 54%; AR vs. ER ≈ 20%	Despite substantial sequence homology among AR, GR, PR, and MR, each receptor maintains high ligand discrimination. Low homology with ER underlies potent androgen–estrogen selectivity.
Conservation of binding pocket residues	Core ligand-binding residues are largely conserved	Conserved residues support steroid recognition, whereas subtle non-conserved differences fine-tune receptor specificity.
Pocket volume and geometry [53,54]	Receptor-specific pocket size and shape	Slight differences in cavity volume and contour restrict or permit accommodation of specific steroid substituents.
Key residue substitutions [53,55]	Example: AR Leu701 vs. GR Met604	Single amino-acid substitutions alter steric constraints and influence ligand orientation and affinity.
Helix 6–7 loop conformation [56,57]	“Open” vs. “closed” conformations	Loop flexibility modulates ligand entry, positioning, and stabilization within the binding pocket.
Non-conserved residues [58,59]	Peripheral pocket residues differ among receptors	Minor variations generate distinct van der Waals contacts, contributing to selective ligand stabilization.
Cross-reactivity of natural steroids [60,61]	Progesterone and cortisol bind multiple receptors.	Structural similarity among steroids allows partial cross-binding, necessitating additional regulatory mechanisms.
Pre-receptor metabolism	Example: 11β-HSD2 confers MR selectivity	Enzymatic inactivation of competing ligands enhances receptor-specific signaling in target tissues.
Synthetic steroid design	Targeted chemical modifications	Rational design exploits structural differences to increase receptor selectivity and reduce off-target effects.
Molecular recognition principles	Steric complementarity, electrostatic interactions, hydrogen bonding, and hydrophobic interactions	Cooperative interactions govern ligand affinity, orientation, and receptor activation state.
Binding mechanism [57,62,63]	Induced-fit conformational changes	Ligand binding reshapes the LBD, stabilizing receptor-specific active or inactive conformations.

**Table 5:** Structure–activity relationships (SAR) of natural and synthetic anabolic–androgenic steroids. The table summarizes key structural features of endogenous androgens, common synthetic modifications, and representative anabolic–androgenic steroid compounds, highlighting their effects on androgen receptor (AR) affinity, agonist potency, pharmacokinetics, and selectivity. Emphasis is placed on how specific chemical modifications of the steroid scaffold—such as 5α-reduction, C17α-alkylation, 19-nor substitution, ring unsaturation, and esterification—modulate anabolic versus androgenic activity, oral bioavailability, tissue selectivity, and the development of tissue-selective androgen receptor modulators (SARMs).

Category	Structural Feature or Compound	SAR Characteristic	Functional/Biological Implication
Natural androgens	Testosterone [64]	Moderate AR affinity (Kd ~1–5 nM)	Baseline androgenic and anabolic activity
	Dihydrotestosterone (DHT) [65]	High AR affinity (Kd ~0.1–0.5 nM)	Potent agonist; forms a more stable AR complex
	5α-reduction [66]	Increases androgen potency	Enhances receptor binding and signaling
	17β-hydroxyl group [67]	Essential functional group	Required for high-affinity AR binding
Synthetic AAS modifications	C17α-alkylation [68]	Increased oral bioavailability	Preserves activity but increases hepatotoxic risk
	C1–C2 double bond [69]	Elevated anabolic/androgenic ratio	Favors anabolic effects
	7α-methylation [70]	Increased receptor potency	Enhances anabolic activity
	19-nor modification [71]	Altered AR selectivity	Reduced androgenic effects relative to anabolic action
	Esterification at 17β-OH [72]	Prodrug formation	Prolongs half-life and sustained release
Representative AAS compounds	Nandrolone (19-nortestosterone) [73]	19-nor structure	High anabolic with reduced androgenic effects
	Stanozolol [50]	C17α-methyl, pyrazole A-ring	Oral activity with distinctive receptor interactions
	Methyltrienolone (R1881) [74]	Multiple unsaturations	Extremely high AR affinity and potency
	Tetrahydrogestrinone (THG) [69]	Designer steroid	High AR affinity; illicit performance enhancement
	Oxandrolone [75]	C17α-methyl, 2-oxa A-ring	Favorable anabolic-to-androgenic ratio
General SAR principles	Steroid nucleus [62]	Conserved tetracyclic scaffold	Required for AR recognition
	3-keto and 17β-hydroxyl groups [76]	Key interaction sites	Critical for agonist binding and activation
	A-ring modifications [70]	Influence electronic and steric properties	Modulate potency and efficacy
	D-ring modifications [61]	Affect receptor selectivity	Shape anabolic versus androgenic profiles
	Bulky substituents (various positions) [67]	Steric hindrance	Can shift agonist activity toward antagonism
Tissue selectivity (SARMs concept)	Differential coregulator recruitment [77]	Context-dependent signaling	Enables tissue-specific anabolic effects
	Tissue-specific gene expression	Selective AR activation	Minimizes androgenic side effects
	Non-steroidal SARMs [78]	Alternative chemical scaffolds	Designed for improved selectivity and safety

**Table 6:** Structural classification of anabolic–androgenic steroids based on key chemical modifications. Anabolic–androgenic steroids are grouped according to defining structural modifications of the steroid nucleus, including C17β esterification, C19 demethylation, 17α-alkylation, A-ring modification, heterocyclic fusion, halogenation, and combined multi-site substitutions. Representative compounds and their principal structural–functional consequences for androgen receptor interaction and pharmacokinetics are summarized.

Structural Class	Defining Structural Modification	Key Carbon Positions Affected	Representative Steroids (Structure Type)	Defining Structural Consequence
Class I—Testosterone and C17β-ester derivatives	Esterification of the native 17β-hydroxyl group	C17β	Testosterone, testosterone propionate, testosterone enanthate, testosterone cypionate	Preserves native androgen receptor (AR) binding geometry; ester chain increases lipophilicity and prolongs release without altering intrinsic receptor affinity
Class II—19-Nor derivatives	Removal of the angular C19 methyl group	C19	Nandrolone, nandrolone phenylpropionate, nandrolone decanoate, norbolethone	Reduces steric bulk near A/B ring junction; alters AR ligand orientation and co-regulator recruitment, producing anabolic bias
Class III—17α-alkylated steroids (oral AAS)	Alkyl substitution at the 17α position (methyl or ethyl)	C17α	Methandrostenolone, Oxandrolone, stanozolol, fluoxymesterone	Blocks oxidative metabolism at C17, conferring oral bioavailability at the cost of hepatotoxic potential
Class IV—A-ring modified/unsaturated steroids	Introduction of double bonds or heteroatoms in the A-ring	C1–C2, C2	Methandrostenolone (Δ^1^), boldenone (Δ^1^), Oxandrolone (2-oxa)	Alters the electronic density and hydrogen-bonding of the A-ring, modifying AR activation profile and anabolic-to-androgenic ratio
Class V—Heterocyclic A-ring steroids	Fusion of a non-steroidal heterocycle to the A-ring	A-ring	Stanozolol (pyrazole-fused A-ring)	Disrupts classical steroid A-ring electronics, resulting in atypical AR docking and reduced androgenic signaling
Class VI—Halogenated steroids	Halogen substitution (typically fluoro)	C9α, C11β	Fluoxymesterone (9α-fluoro)	Increases receptor binding affinity and residence time through enhanced hydrophobic and electrostatic interactions
Class VII—Combined multi-modified steroids	Multiple concurrent modifications (e.g., 17α-alkyl + unsaturation + halogenation)	C1, C9, C17	Fluoxymesterone, methyltrienolone	Synergistic enhancement of potency, metabolic stability, and non-genomic signaling bias

**Table 7:** C17 substitutions in androgenic steroids and their structural–functional consequences. This table summarizes the principal classes of chemical substitutions at the C17 position of androgenic and anabolic–androgenic steroids, highlighting representative compounds and their functional implications. Modifications at C17 critically determine the stability of androgen receptor (AR) binding, metabolic susceptibility, pharmacokinetics, and the route of administration. Native 17β-hydroxyl and 17-keto steroids exhibit limited oral bioavailability, whereas 17α-alkylation confers metabolic resistance at the cost of hepatotoxic risk. In contrast, 17β-esterification produces long-acting injectable prodrugs without altering intrinsic AR affinity.

C17 Substitution Class	C17 Functional Group	Representative Steroids
17β-Hydroxyl	17β–OH	Testosterone; Dihydrotestosterone (DHT); 5α-Androstanediol
17-Keto	17–C=O	Androstenedione; Androstanedione; Dehydroepiandrosterone (DHEA)
17α-Alkyl	17α–CH_3_, 17α–C_2_H_5_	Methyltestosterone; Oxandrolone; Stanozolol; Fluoxymesterone; Metandienone
17β-Esterified hydroxyl	Fatty acid esters (acetate, propionate, enanthate, cypionate, undecanoate)	Testosterone acetate; Testosterone enanthate; Testosterone cypionate; Testosterone undecanoate; Boldenone undecylenate
17-Ether derivatives	17–OR (alkyl ether)	Testosterone 17-methyl ether; Testosterone 17-ethyl ether
17-Hydrogen (unsubstituted)	–H	Androstane; 5α-Androstane
Bulky or heterocyclic substituents	Heterocycles or extended side chains	Danazol; modified nandrolone derivatives
17β-Alkyl (non-ester)	Small alkyl at 17β	Nandrolone derivatives
Polar synthetic substituents	Carboxamide or polar side chains	Experimental AR ligands

## Data Availability

Data are availabe on the request.
